# Comparative Analysis of Compatibility Effects on Invigorating Blood Circulation for Cyperi Rhizoma Series of Herb Pairs Using Untargeted Metabolomics

**DOI:** 10.3389/fphar.2017.00677

**Published:** 2017-09-26

**Authors:** Pei Liu, Er-Xin Shang, Yue Zhu, Jin-Gao Yu, Da-Wei Qian, Jin-Ao Duan

**Affiliations:** Jiangsu Key Laboratory for High Technology Research of TCM Formulae, Jiangsu Collaborative Innovation Center of Chinese Medicinal Resources Industrialization, National and Local Collaborative Engineering Center of Chinese Medicinal Resources Industrialization and Formulae Innovative Medicine, Nanjing University of Chinese Medicine, Nanjing, China

**Keywords:** herb pairs, Cyperi Rhizoma, compatibility, acute blood stasis, metabolomics, principal component analysis

## Abstract

The mutual-assistance compatibility of Cyperi Rhizoma (Xiangfu, XF) and Angelicae Sinensis Radix (Danggui, DG), Chuanxiong Rhizoma (Chuanxiong, CX), Paeoniae Radix Alba (Baishao, BS), or Corydalis Rhizoma (Yanhusuo, YH), found in a traditional Chinese medicine (TCM) named Xiang-Fu-Si-Wu Decoction (XFSWD), can produce synergistic and promoting blood effects. Nowadays, XFSWD has been proved to be effective in activating blood circulation and dissipating blood stasis. However, the role of the herb pairs synergistic effects in the formula were poorly understood. In order to quantitatively assess the compatibility effects of herb pairs, mass spectrometry-based untargeted metabolomics studies were performed. The plasma and urine metabolic profiles of acute blood stasis rats induced by adrenaline hydrochloride and ice water and administered with Cyperi Rhizoma—Angelicae Sinensis Radix (XD), Cyperi Rhizoma—Chuanxiong Rhizoma (XC), Cyperi Rhizoma—Paeoniae Radix Alba (XB), Cyperi Rhizoma—Corydalis Rhizoma (XY) were compared. Relative peak area of identified metabolites was calculated and principal component analysis (PCA) score plot from the potential markers was used to visualize the overall differences. Then, the metabolites results were used with biochemistry indicators and genes expression values as parameters to quantitatively evaluate the compatibility effects of XF series of herb pairs by PCA and correlation analysis. The collective results indicated that the four XF herb pairs regulated glycerophospholipid metabolism, steroid hormone biosynthesis and arachidonic acid metabolism pathway. XD was more prominent in regulating the blood stasis during the four XF herb pairs. This study demonstrated that metabolomics was a useful tool to efficacy evaluation and compatibility effects of TCM elucidation.

## Introduction

Cyperi Rhizoma (XF), the rhizome of *Cyperus rotundus* L., has commonly been used for the treatment of gynecological or emotional disorders in traditional medicine (Kim et al., 2013). In clinical practice, the compatibility application between XF and other herb medicine, such as Angelicae Sinensis Radix [DG, the radix of *Angelica sinensis* (Oliv.) Diels], Chuanxiong Rhizoma (CX, the rhizome of *Ligusticum chuanxiong* Hort.), Paeoniae Radix Alba (BS, the radix of *Paeonia lactiflora* Pall.), Corydalis Rhizoma (YH, the rhizome of *Corydalis yanhusuo* W.T. Wang), can be found in Xiang-Fu-Si-Wu Decoction (XFSWD) of *Buzhiyi Biyao*, which is an ancient and classic formula with the activating blood circulation and dissipating blood stasis effects. The formula contains numerous and diverse constituents including phthalide, alkaloids, terpene lactones, etc. The metabolomics study in animals and patients with blood stasis syndrome has given evidence that the formula prevents and benefits many disorders throughout the entire body (Liu et al., 2011a, 2013a,b,c, 2014a,b).

XF is a core medicine in XFSWD, which acts as an indispensable part and aimed at regulating Qi and blood. DG could invigorate the circulation of blood, which has been called “female ginseng” (Li et al., 2015). CX is also a medicine for regulating blood circulation and promoting Qi circulation. Phthalides are usually considered to be the active constituents of DG and CX with the effects of inhibiting vasoconstriction, anti-proliferation, anti-oxidation and anti-inflammation (Han B. et al., 2016). BS mainly contains terpene lactones, with claims of antispasmodic, tonic, astringent and analgesic properties (Shu et al., 2014). YH, which famous for its analgesic alkaloid compounds, is widely used to promote blood circulation and relieve pain (Liao, D. et al., 2016). The four herb pairs, including Cyperi Rhizoma—Angelicae Sinensis Radix (XD), Cyperi Rhizoma—Chuanxiong Rhizoma (XC), Cyperi Rhizoma—Paeoniae Radix Alba (XB), Cyperi Rhizoma—Corydalis Rhizoma (XY) were the basic units of XFSWD for its effect of invigorating the circulation of blood. These four herb pairs have the higher occurrence frequency than others in the classical prescriptions by a data mining method (Liu et al., 2011b). They played a key role in revealing the composition and compatibility of this complex formula.

To illustrate the synergistic mechanisms of Cyperi Rhizoma series of herb pairs for the mutual assistance compatibility, metabolomics methods were introduced. As we know, metabolomics has brought much excitement to the field of life sciences as a potential translational tool. The method has been adopted to research the complex problems of traditional Chinese medicine (TCM) (Cui et al., 2017; Wang et al., 2017). As an “omic” science, metabolomics have rich information concerning all the metabolites in bio-fluids, which could indicate early biological changes to the host due to perturbations in disease. The metabolic markers found by metabolomics methods could detect early stage disease and help to monitor treatment response. Those reliable and sensitive biomarkers could track the disease and develop therapies during the course of treatment (Zhang A. et al., 2015, 2016). Metabolomics based on mass spectrometry (MS) was a powerful technique enables the parallel assessment of the levels of a broad range of metabolites. Till now, the mechanism of therapeutic effects and compatibility characteristic of formulae and herb pairs was elucidated by the technique of metabolomics (Dong et al., 2015; Chu et al., 2016; Zhang, Q. Y. et al., 2016; Zhao et al., 2016; Shen et al., 2017).

Comprehensive metabolomics measurements in combination with chemometrics methods are potentially very useful for evaluation of the therapeutic effects of TCM (Liu et al., 2016). Person correlation coefficient analysis and hierarchical cluster analysis (HCA) were usually used to analyze the metabolite-metabolite correlation among identified metabolites (Rao et al., 2016), while principal components analysis (PCA), partial least squares discriminant analysis (PLS-DA), orthogonal partial least-squared discriminant analysis (OPLS-DA), as well as univariate statistical methods, such as *T*-test and analysis of variance (ANOVA) are professional approaches to analyze and maximize information retrieval from complex raw data (Wang et al., 2017). PCA can summarize the information in an experimental data set using a small number of orthogonal latent variables obtained by searching the direction of maximum variance in the data set (Huang et al., 2016).

In this study, in order to assess the compatibility effects and characteristic of Cyperi Rhizoma series of herb pairs in XFSWD on the treatment of gynecological blood stasis syndrome quantitatively, liquid chromatograph-mass spectrometer based untargeted metabolomics studies were performed. The efficacy effects of XD, XC, XB, and XY pairs on the treatment of acute blood-stasis model rats were evaluated. The overall perturbations observed in plasma and urine metabolic profiles were compared. Two parameters, Pearson correlation coefficient and PCA score, were used to describe the inner relationship of pharmacological bioactivity and biochemical mechanism, quantitatively evaluate the compatibility trait of the four herb pairs from the aspect of global metabolic profile, respectively.

## Materials and methods

### Chemicals and reagents

The acetonitrile, methanol, and formic acid were all of HPLC grade and purchased from Merck (Darmstadt, Germany). Deionized water was purified by a Millipore Q5 purification system (MerckMillipore, Bedford, MA, USA). Other reagent solutions were of analytical grade (Sinopharm Chemical Reagent Co. Ltd., Shanghai, China).

Reference standards of lysoPCs was obtained from Avanti (USA), estrone was obtained from the National Institutefor the Control of Pharmaceutical and Biological Products (Beijing, China), arachidonic acid was obtained from Aladin (Shanghai, China). Adrenaline hydrochloride was purchased from Harvest pharmaceutical Co. Ltd., Shanghai (batch number: 10150103, Shanghai, China). Aspirin enteric-coated tablets were obtained from Bayer Schering Pharma (batch number: BJ16177, Beijing, China). Adenosine diphosphate (ADP) and thrombin were obtained from Nanjin Jiancheng biotechnology co. LTD (batch number: 20160219, 20160328, Nangjing, China).

The crude drug: Cyperi Rhizoma, Angelicae Sinensis Radix, Chuanxiong Rhizoma, Paeoniae Radix Alba, Corydalis Rhizoma, were purchased from Nanjing Medicinal Material Company and authenticated by the corresponding author. They were within the qualitative and quantitative stipulation of Chinese Pharmacopoeia (2015). The voucher specimens (NO. NJUCM 201603201-2016032005) were kept in the Herbarium of Nanjing University of Chinese Medicine, Nanjing, PR China.

In XFSWD, the dose of XF, DG, CX, BS, and YH was 4.5, 9, 4.5, 4.5, and 4.5 g, respectively. To keep the four herb pairs at the same level for comparison, the ratio of 1:1 was chosen. A total 500 g mixed pieces of Cyperi Rhizoma—Angelicae Sinensis Radix (XD) (1: 1, w/w) were extracted with boiling water (1: 8) for twice, 2 h for each time, filtered through gauze, respectively. The two filtrates were merged and evaporated with rotary evaporation under vacuum at 50°C, thus, XD extracts were obtained. Cyperi Rhizoma—Chuanxiong Rhizoma (XC), Cyperi Rhizoma—Paeoniae Radix Alba (XB), Cyperi Rhizoma—Corydalis Rhizoma (XY) were extracted through the same procedure, and then XC, XB, XY extracts could also be obtained.

### Animal study and sampling

All experiments were performed with female SD rats (200 ± 20 g), obtained from Shanghai Jiesijie laboratory animal company (Shanghai, China). The protocol was approved by the Animal Ethics Committee of Nanjing University of Chinese Medicine. The investigation was conducted in accordance with the ethical principles of animal use and care. After 1 week of acclimatization, the rats were randomly divided into seven groups with eight rats in each: the normal, model, aspirin, XD, XC, XB, and XY groups. The rats in model, aspirin, XD, XC, XB, and XY groups were hypodermically injected with adrenaline hydrochloride twice at dose of 0.8 mg/kg on day 3, time interval for 4 h. Two hour after the first hypodermic injection with adrenaline hydrochloride, the rats were put in 0–2°C ice water to swim in 4 min, causing acute blood stasis rats model (Li et al., 2015). The rats in XD, XC, XB, and XY groups were intragastrically given XD, XC, XB, and XY extracts at a dose of 4.86 g/kg (4.86 g crude herbs per 1 kg rat body weight). The aspirin group rats were oral administration with aspirin enteric-coated tablets dispersed homogeneously in water at a dose of 9 mg/kg. The animal dose of XD, XC, XB, and XY extracts was extrapolated from the human daily dose, using the body surface area normalization method. The formula for dose translation was as follows: human dose of crude herbs in clinic × 0.018/200 × 1,000 × the multiple of clinical equivalency dose. The dose of XD, XC, XB, and XY extracts was equivalent to three times of the adult daily dose herb pair XD, XC, XB, and XY (18 g, from XFSWD in which Cyperi Rhizoma were 9 g) crude herbs based on the TCM prescription. The normal and model groups were intragastrically given the same volume of saline solution. All animals were administered by oral gavage two times each day for continuous seven times.

All rats were housed in metabolic cages (one per cage) after the last time of subcutaneous injection of adrenaline hydrochloride in model and administration groups rats. They were free to drink water but no diet for 12 h. The next morning, urine samples were centrifuged at 3,000 rpm for 10 min immediately. Then, the supernatants were separated and stored at −80°C until analysis. After half an hour of the last time of administration, 10% choral hydrate (350 mg/kg intraperitoneal injection) was used for anesthesia. Blood samples of rats were collected by carotid artery intubationapproach into 10 mL Eppendorf centrifuge tubes (Sodium citrate ACTS as an anticoagulant, 109 mmol/L). Then, 800 μL of whole blood were used to measure whole blood viscosity. The rest of the whole blood samples were immediately centrifuged at 3,000 rpm for 10 min. 200 μL supernatants of plasma samples were separated and stored at −80°C for UPLC-MS/MS analysis. Eight hundred microliters of supernatants of plasma samples were used to the plasma viscosity (PV) test through LG-R-80 B computer blood viscosity tester. The rest plasma samples were used to ADP-induced platelet maximum aggregation (PMAR) and thrombin time (TT) test.

The tissues' samples, including uterus and ovary, were excised for endocrine gene expression test. They were thoroughly rinsed with physiological saline solution, and then blotted dry with filter paper and stored at −80°C.

### Sample preparation and metabolomic analysis

Sample preparation was based on our previous studies (as shown in Supplementary Information). All tested samples were analyzed randomly. LC-MS analysis was performed on a Waters ACQUITY UPLC system (Waters Corporation, Milford, USA). Acquity UPLC BEH-C18 column (2.1 × 100 mm, 1.7 μm) was applied for all analyses. The mobile phase was composed of A (0.1% formic acid, v/v) and B (acetonitrile).

For plasma analysis, the mobile phase with a linear gradient elution: 0–2 min, A: 95–70%; 2–4 min, A: 70%; 4–6 min, A: 70–40%; 6–13 min, A: 40%; 13–19 min, A: 40–3%; 19–20 min, A: 3–95%. For urine analysis, the mobile phase with a linear gradient elution: 0–4 min, A: 95−80%; 4–11 min, A: 80–60%; 11–13 min, A: 60–10%; 13–14 min, A: 10–95%. The flow rate of the mobile phase was 0.4 mL/min, and the column temperature was maintained at 35°C.

MS was performed on a Synapt™ Q-TOF (Waters, Manchester, UK). The instrument was operated by using an electrospray ionization (ESI) source in negative mode. The ionization source conditions were as follows: capillary voltage of 3.0 kV, source temperature of 120°C and desolvation temperature of 400°C. The sampling cone voltage was set at 30 V, extraction cone was 4.0 V, trap collision energy was 6.0 V, transfer collision energy 4.0 V, trap gas flow was 1.50 mL/min, ion energy was at 1.0 V. Nitrogen and argon were used as cone and collision gases, respectively. The cone and desolvation gas flow were 50 and 600 L/h, respectively. The scan time of 0.3 s was used throughout with interval scan time of 0.02 s and with collision energy of 6 eV. The MS data was collected from m/z 100 to 1,000 Da in negative ion in centroid mode.

Data acquisition and processing were performed by using Masslynx™ v 4.1 (Waters Corp.). All data were normalized to the summed total ion intensity per chromatogram, and the resultant data matrices were introduced to SIMICA-P 15.0 software (Umetrics AB, Umeå, Sweden) for PCA and OPLS-DA. All variables obtained from UPLC-MS/MS data sets were UV and scaled to Pareto variance. The OPLS-DA score plots were described by the cross-validation parameter R^2^Y and Q^2^, which represents the total explained variation for the X matrix and the predictability of the model, respectively. S-plot and variable importance in the projection (VIP) were used for the selection of potential markers. Variables with VIP values larger than 1 were chosen as more important for the explanation of Y (response).

### *q*PCR analysis

To determine the relative mRNA expression levels of endocrine related genes, total RNA was isolated from ovary and uterus samples by Trizol reagent (Invitrogen, Carlsbad, CA) according to the manufacture's instruction. Up to 1 μg of total RNAs isolated from tissues were reverse transcribed by using PrimeScript™ RT reagent Kit with gDNA Eraser (Takara, Japan) according to the manufacture's instruction, and the cDNA of each sample was obtained. Real-time quantitative PCR was performed on equal amounts of cDNA by using SYBR Green Master mix with Rox reference dye (Roche, Germany). The SYBR green signal was detected by the ABI PRISM 7500 sequence detection system (Applied Bio systems). The relative amounts of each mRNA were calculated using the 2^−ΔΔCT^ method (Livak and Schmittgen, 2001). mRNA levels are expressed as fold changes relative to expression of β-actin. The primers were as follows: 5′-TGA AGC GGC AAA TCT CTG AAC-3′ (sense primer, S) and 5′-CAT CTG GCT TTG GTG AGC AG-3′ (anti-primer, AS) for follicle-stimulating hormone (FSH, 210 bp, NM_199237.1); 5″-TCT GTT CAC CCA AGA CAC TCC-3′ (S) and 5′-AGG TAG AGC CCC ATG CAA AAG-3′ (AS) for luteinizing hormone (LH, 232 bp, NM_012978.1); 5′-CTA GCC TAC ATG GAG GGA GTG-3′ (S) and 5′-CCA CAC TTC AGA GGC AGA GAA-3′ (AS) for androgen (AD, 233 bp, NM_012502.1); 5′-GAC ATG AGA GCT GCC AAC CT-3′ (S), and 5′-GGC ACT CTC TTT GCC CAG TT-3′ (AS) for estrogen (Es, 242 bp, NM_012689.1).

### Statistical analysis

The experimental data were presented as mean ± SD. Statistical significance was assessed by ANOVA test and paired-sample *t*-test was adopted to test normal distribution by SPSS v18.0 (SPSS, Inc., Chicago, IL, USA). In all experiments, confidence level was set at 95% to determine the significance of difference (*p* < 0.05).

Pearson correlation coefficients were calculated with SPSS v18.0 (IBM SPSS, USA). The correlation heat-map was built and optimized with HemI software (Deng et al., 2014). The correlation coefficient is always between −1 and +1. The closer the correlation is to ±1, the closer to a perfect linear relationship.

The results of a PCA are usually discussed in terms of component scores. Consider a data matrix, X, where each of the *n* rows represents different samples, and each of the *p*-columns gives the results tested factors. A set of *p*-dimensional vectors of loadings (α_*ij*_) map each row vector (x_*i*_) of X to a new vector of the principal component scores (F_*j*_), given by *F*_*j*_ = α_1*j*_x_1_+α_2*j*_x_2_+…+α_*ij*_x_*i*_, for *i* = 1, 2, …, n, *j* = 1, 2, …, m. The full principal components score (F) decomposition of X can therefore be given as *F* = ρ_1_F_1_+ρ_2_F_2_+…ρ_*j*_F_j_, where ρ_*j*_ is the *j*th eigenvector of F_*j*_ (Abdi and Williams, 2010).

## Results

### Hemorheological indexes and pathological section

The whole blood viscosity (WBV), PV, ADP-induced PMAR and TT were usually considered to assess blood stasis degree and treatment efficacy (Zhang, X. T. et al., 2015). Compared to the normal group, WBV, PV, and PMAR levels in the model group (acute blood stasis model rats) were significantly increased; while TT was significantly shortened (*p* < 0.01). Compared to the model group, the positive medicine group (Aspirin) of WBV(5S^−1^), WBV(1S^−1^), PV, and PMAR were significantly reduced, TT was significantly lengthened (*p* < 0.01). In the four treatment groups, only XD group of WBV, PV, PMAR, and TT, which values tended to the normal group, showed significantly change. Compared with model group, The XC, XB, and XY group of test indexes had some improvement with no significantly change, except PV (Table 1).

**Table 1:** The whole blood viscosity (WBV), plasma viscosity (PV), ADP-induced platelet maximum aggregation (PMAR) and thrombin time (TT), changes of treatment groups and normal group (*x* ± s, *n* = 8).

**Group**	**WBV (ηb/mPa•s)**				**PV (ηb/mPa•s)**	**PMAR (%)**	**TT (s)**
	**200 S^−1^**	**30 S^−1^**	**5 S^−1^**	**1 S^−1^**	**200 S^−1^**		
Normal	4.51 ± 0.50	5.55 ± 0.63	8.44 ± 1.05	16.45 ± 2.42	1.58 ± 0.09	15.67 ± 1.38	27.01 ± 1.53
Model	5.26 ± 0.84^#^	6.49 ± 1.16^#^	10.28 ± 1.84^#^	20.67 ± 4.22^#^	2.73 ± 0.11^##^	17.70 ± 1.25^#^	24.23 ± 2.39^##^
Aspirin	4.78 ± 0.48	5.96 ± 0.65	8.91 ± 1.09^*^	17.12 ± 2.44^*^	1.60 ± 0.09^**^	15.83 ± 1.44^*^	26.69 ± 1.76^*^
XD	4.45 ± 0.64^*^	5.62 ± 0.78^*^	8.47 ± 1.39^*^	16.79 ± 3.14^*^	1.60 ± 0.09^**^	15.93 ± 1.23^*^	26.50 ± 1.61^*^
XC	5.14 ± 0.55	6.37 ± 0.74	9.83 ± 1.37	19.45 ± 3.10	1.66 ± 0.08^**^	16.23 ± 1.58	25.59 ± 1.64
XB	5.02 ± 1.17	6.36 ± 1.55	9.71 ± 2.70	19.06 ± 6.19	1.65 ± 0.10^**^	16.65 ± 1.83	25.71 ± 1.68
XY	5.27 ± 0.24	6.58 ± 0.35	10.25 ± 0.72	20.55 ± 1.93	1.71 ± 0.11^**^	17.20 ± 2.33	25.91 ± 1.72

# *p < 0.05*,

## *p < 0.01 compared to the normal group*.

* *p < 0.05*,

** *p < 0.01 compared to the model group*.

HE staining of liver, ovary and uterus sections revealed that there was a statistically insignificant trend for pathological change in acute blood stasis model rats and the XD, XC, XB, XY treatment rats (Figure S1).

### Multivariate data analysis for blood plasma and urine

Plasma and urine from seven groups' rats (normal, model, aspirin, XD, XC, XB, and XY) were used to the metabonomics analysis by UPLC-Q-TOF/MS. Representative plasma and urine spectra are displayed in Figures S2, S3.

**Figure 1:**
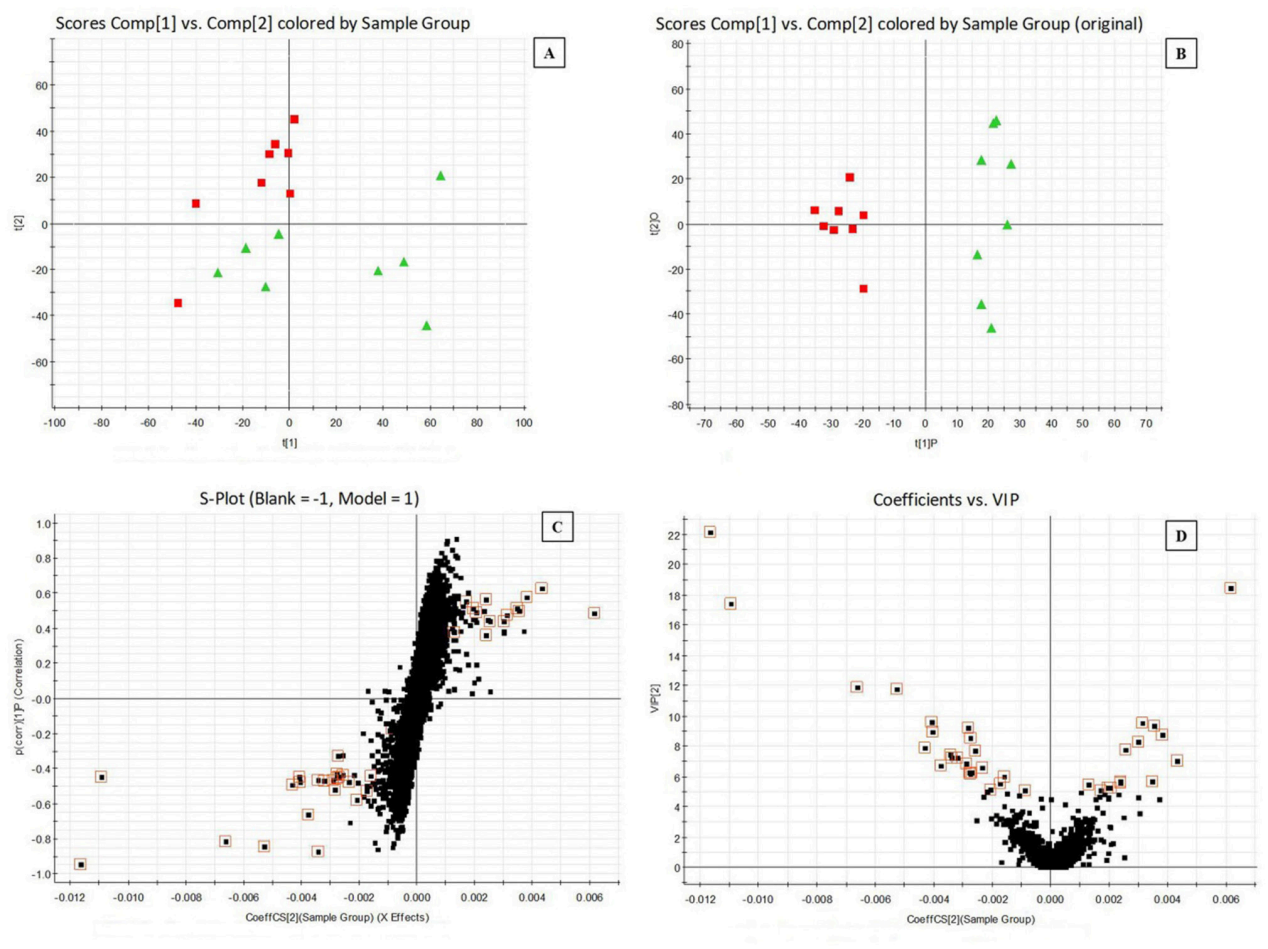
Principal components analysis (PCA) scores plots for plasma samples of **(A)** normal group (red 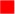) vs. model group (green 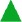) and orthogonal partial least-squared discriminant analysis (OPLS-DA) scores plots of **(B)** normal group (red 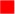) vs. model group (green 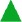). **(C)** S-plot of OPLS-DA and **(D)** VIP-plot of OPLS-DA in negative mode.

**Figure 2:**
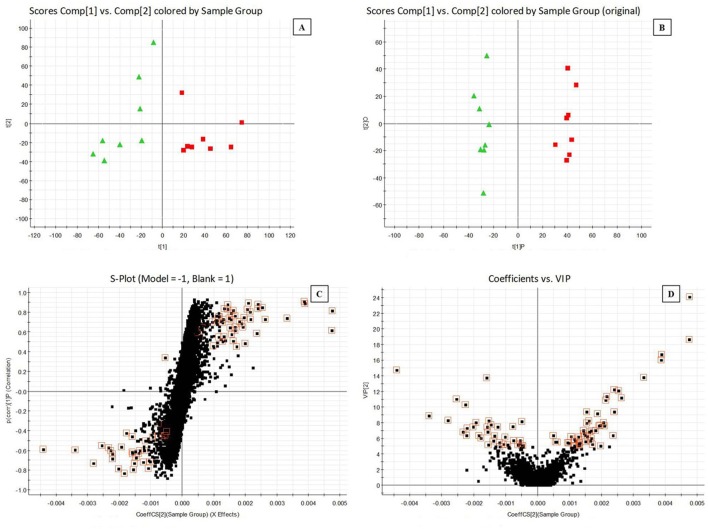
Principal components analysis (PCA) scores plots for urine samples of **(A)** normal group (red 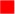) vs. model group (green 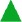) and orthogonal partial least-squared discriminant analysis (OPLS-DA) scores plots of **(B)** normal group (red 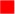) vs. model group (green 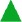). **(C)** S-plot of OPLS-DA and **(D)** VIP-plot of OPLS-DA in negative mode.

Clear clustering of normal vs. model samples was showed in PCA scores plots (Figures 1A, 2A), which demonstrated the blood stasis pathological process caused by subcutaneous injection of adrenaline hydrochloride and ice water bath changed in biofluid metablites. Supervised OPLS-DA model was built to find potential markers for the blood stasis pathological process. OPLS-DA distinguished the normal and model cohorts with 100% sensitivity and no <95% specificity using a leave one out algorithm. R^2^Y of this OPLS-DA model was 0.957 (plasma samples) and 0.983 (urine samples) respectively. Q^2^ was 0.630 (plasma samples) and 0.799 (urine samples) (Figures 1B, 2B). Using a full external validation paradigm, discriminatory sensitivity was 100% and specificity no <90%.

Potential metabolites responsible for the separation between normal and model samples could be selected from the S-plot and VIP-value plot (Figures 1C,D, 2C,D). The retention time, precise molecular mass and MS/MS data for the structural identification of metabolites were obtained from the UPLC-Q-TOF/MS analysis platform according to the reference method (Liu et al., 2013c; Li et al., 2015). In this experiment, the variables were selected as candidates when their VIP-values were larger than five. The measurement error was with 10 mDa. The presumed molecular formula was searched in METLIN (https://metlin.scripps.edu/) and other databases to identify the possible chemical constitutions. MS/MS data were screened to determine the potential structures. Finally, reference compounds, MS/MS spectrum in online databases and literatures data were used to confirm some of the identified markers.

According to the protocol detailed above, 23 endogenous metabolites, such as lysoPE(22:4/0:0), trihydroxycholan-24-oic acid, lysoPC(17:0), lysoPE(20:2/0:0), lysoPE (24:6/0:0), taurodeoxycholic acid, arachidonic acid, tauroursocholic acid, lysoPE(22:1/0:0), chimonanthine, PG(10:0/10:0), cofaryloside, PC(19:3/0:0), lysoPE(0:0/18:0), indoxylsulfuric acid, estrone 3-glucuronide, 5-(4-acetoxybut-1-ynyl)-2, 2′-bithiophene, polyethylene oxidized, succinoadenosine, 17-hydroxypregnenolone sulfate, uralenneoside, 2, 8-dihydroxyquinoline-beta-D-glucuronide, and dihydrocaffeic acid 3-sulfate, contributing to the separation of the normal and model rats were detected in the samples (Table 2). Pathway analysis was performed with MetaboAnalyst. The pathway with the impact-value threshold above 0.10 was regarded as potential target pathway (Wang et al., 2012; Xia and Wishart, 2016). These identified metabolites mainly participated in three pathways, including arachidonic acid metabolism, steroid hormone biosynthesis and glycerophospholipid metabolism as the pathway with the impact value threshold above 0.10 (Figure S4).

**Table 2:** Potential metabolites selected and identified in rats urine and plasma by UPLC-Q-TOF-MS/MS.

**No**.	***t*_R_(min)**	**m/z**	**VIP value**	**Mass accuracy^a^ (ppm)**	**MS/MS**	**Metabolites**	**Source**
PM1	8.23	528.3094	22.13	−0.38	387.3 [M-H-C_2_H_8_NO_4_P]^−^ 271.2 [M-H-C_7_H_16_NO_7_P]^−^	lysoPE(22:4/0:0)	plasma
PM2	6.29	407.2803	18.44	0	251.1 [M-H-C_9_H_16_O_2_]^−^	trihydroxycholan-24-oic acid	plasma
PM3	13.82	508.3417	17.41	1.6	326.2 [M-H-C_5_H_13_NO_4_P]^−^ 282.3 [M-H-C_7_H_17_NO_5_P]^−^	lysoPC(17:0)^b^	plasma
PM4	7.91	504.3098	9.60	0.4	474.3 [M-H-CH_4_N]^−^ 303.2 [M-H-C_5_H_16_NO_5_P]^−^	lysoPE(20:2/0:0)	plasma
PM5	8.13	552.3085	9.20	−2.0	509.3 [M-H-C_2_H_5_N]^−^ 411.3 [M-H-C_2_H_7_NO_4_P]^−^	lysoPE(24:6/0:0)	plasma
PM6	5.67	498.2894	8.72	−0.2	413.2 [M-H-H_5_O_3_S]^−^ 331.2 [M-H-C_4_H_9_NO_4_S]^−^	taurodeoxycholic acid	plasma
PM7	16.8	303.2340	8.54	3.3	259.2 [M-COO]^−^ 205.2 [M-H-C_7_H_14_]^−^	arachidonic acid^b^	plasma
PM8	4.55	514.2839	8.26	−1.0	413.2 [M-H-H_5_O_4_S]^−^ 365.2 [M-H-C_3_H_3_NO_4_S]^−^	tauroursocholic acid	plasma
PM9	14.6	534.3562	6.60	−0.6	508.3 [M-H-CN]^−^ 337.2 [M-H-C_5_H_12_NO_5_P]^−^	lysoPE(22:1/0:0)	plasma
PM10	6.57	345.2083	5.66	−0.6	315.2 [M-H-2CH_3_]^−^	chimonanthine	plasma
PM11	8.13	553.3133	5.55	−2.5	476.3 [M-H-C_3_H_9_O_2_]^−^ 327.2 [M-H-C_10_H_23_O_2_-3OH]^−^	PG(10:0/10:0)	plasma
PM12	3.2	513.2733	5.25	5.5	333.0 [M-H-C_6_H_12_O_6_]^−^ 179.0 [M-H-C_20_H_30_O_4_]^−^	cofaryloside	plasma
PM13	9.09	530.3250	5.15	−0.4	362.2 [M-H-C_5_H_15_NO_3_P]^−^ 255.2 [M-H-C_19_H_31_O]^−^	PC(19:3/0:0)	plasma
PM14	8.7	480.3093	5.04	−1.2	340.2 [M-H-C_2_H_7_NO_4_P]^−^ 255.2 [M-H-C_16_H_33_]^−^	lysoPE(0:0/18:0)	plasma
PM15	3.33	212.0001	18.67	8.0	132.0 [M-H-SO_3_]^−^	indoxylsulfuric acid	urine
PM16	4.1	445.1885	10.30	3.8	253.0 [M-H-C_6_H_8_O_7_]^−^ 192.1 [M-H-C_18_H_21_O]^−^	estrone 3-glucuronide^b^	urine
PM17	3.17	275.0211	8.88	1.8	230.1 [M-H-C_2_H_5_O]^−^ 203.0 [M-H-C_3_H_4_O_2_]^−^	5-(4-acetoxybut-1-ynyl)-2,2′-bithiophene	urine
PM18	6.61	243.1217	8.23	−7.4	213.1 [M-H-CH_2_O]^−^ 201.0 [M-H-C_2_H_2_O]^−^	polyethylene oxidized	urine
PM19	1.8	382.0993	7.44	−2.9	249.0 [M-H-C_5_H_9_O_4_]^−^ 265.1 [M-H-C_4_H_5_O_4_]^−^	succinoadenosine	urine
PM20	10.24	411.1842	6.19	−0.7	329.2 [M-H-H_2_SO_3_]^−^	17-hydroxypregnenolone sulfate	urine
PM21	1.44	321.0366	6.03	−1.6	194.0 [M-Cl-C_6_H_5_O]^−^	uralenneoside	urine
PM22	3.21	336.0707	5.99	−5.4	173.0 [M-H-C_9_H_9_NO_2_]^−^	2,8-dihydroxyquinoline-beta-D-glucuronide	urine
PM23	2.27	261.0062	5.54	−4.6	181.0 [M-H-SO_3_]^−^ 163.0 [M-H-H_2_SO_4_]^−^	dihydrocaffeic acid 3-sulfate	urine

a *Mass accuracy (ppm) was calculated according to the exact mass on the Pubchem website*.

b *Confirmed by standard samples*.

### Metabolic changes correlated with cyperi rhizoma sseries of herb pairs intervention

**Figure 3:**
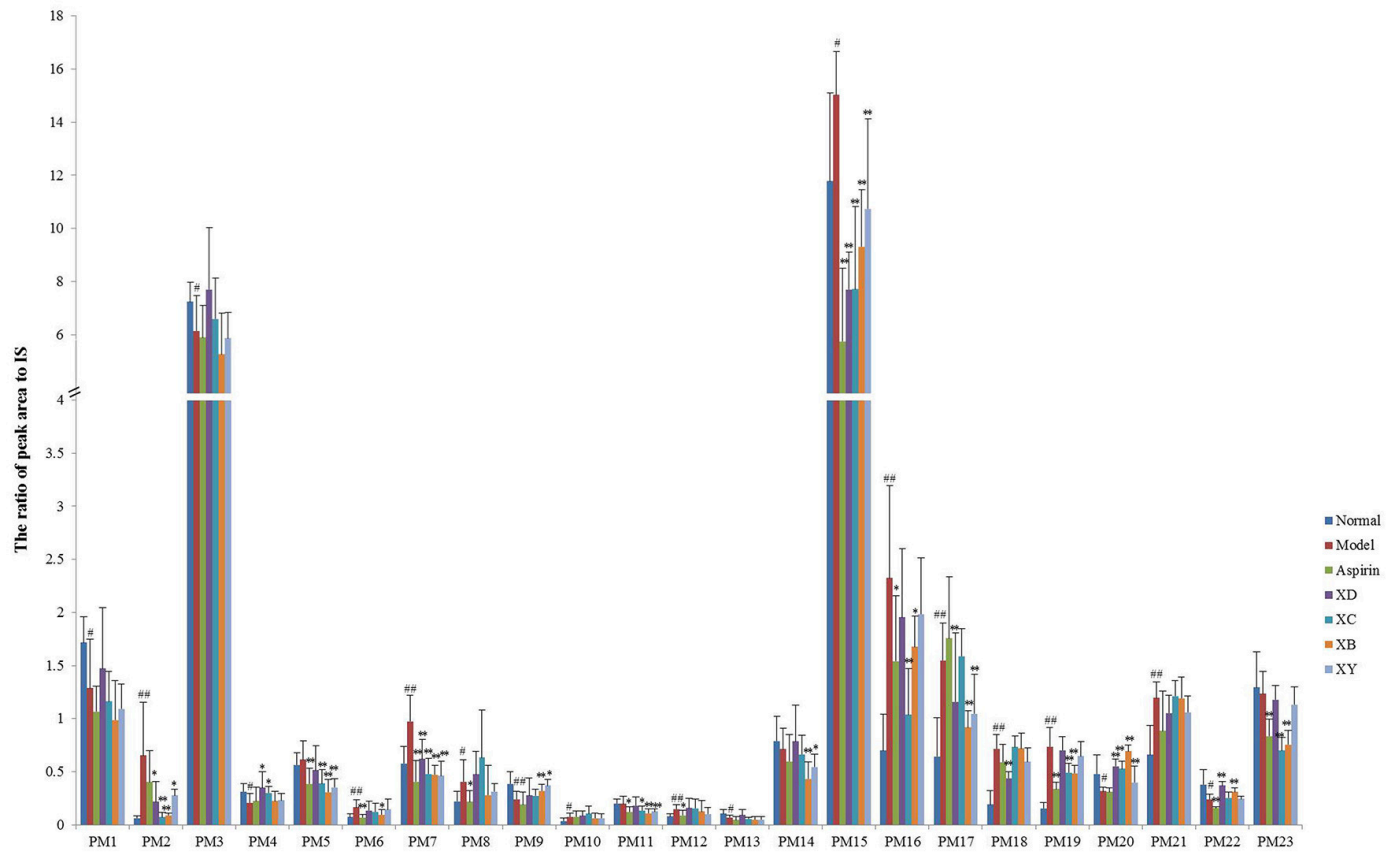
Changes in the relative peak area (vs. IS) of target metabolites identified in plasma and urine (mean ± SD, *n* = 8). ^#^*p* < 0.05, ^##^*p* < 0.01 compared to the normal group. ^*^p < 0.05, ^**^p < 0.01 compared to the model group. PM1, lysoPE(22:4/0:0); PM2, trihydroxycholan-24-oic acid; PM3, lysoPC(17:0); PM4, lysoPE(20:2/0:0); PM5, lysoPE(24:6/0:0); PM6, taurodeoxycholic acid; PM7, arachidonic acid; PM8, tauroursocholic acid; PM9, lysoPE(22:1/0:0); PM10, chimonanthine; PM11, PG(10:0/10:0); PM12, cofaryloside; PM13, PC(19:3/0:0); PM14, lysoPE(0:0/18:0); PM15, indoxylsulfuric acid; PM16, estrone 3-glucuronide; PM17, 5-(4-acetoxybut-1-ynyl)-2,2′-bithiophene; PM18, polyethylene oxidized; PM19, succinoadenosine; PM20, 17-hydroxypregnenolone sulfate; PM21, uralenneoside; PM22, 2,8-dihydroxyquinoline-beta-D-glucuronide; PM23, dihydrocaffeic acid 3-sulfate.

To determine whether different herb pairs of Cyperi Rhizoma was possible to influence metabolic pattern of the acute blood stasis model rats, the above 23 metabolites were semi-quantified by calculating peak area. ANOVA and paired-sample *t*-test were used to compare the relative concentrations (the ratio of peak area to IS) of 23 metabolites in normal, model group, aspirin group, XD group, XC group, XB group, and XY group (Figure 3, Table S1).

LysoPE(24:6), PG(10:0/10:0), lysoPE(0:0/18:0), and dihydrocaffeic acid 3-sulfate was not considered as the markers. For its concentration showed no significant differences between normal and model group based on the relative concentrations data. Compared with the normal group, the concentration of trihydroxycholan-24-oic acid, taurodeoxycholic acid, arachidonic acid, tauroursocholic acid, chimonanthine, cofaryloside, indoxylsulfuric acid, estrone 3-glucuronide, 5-(4-acetoxybut-1-ynyl)-2, 2′-bithiophene, polyethylene oxidized, succinoadenosine, and uralenneoside increased significantly in model group. LysoPE(22:4/0:0), LysoPC(17:0), lysoPE(20:2/0:0), lysoPE(22:1/0:0), PC(19:3/0:0), 17-hydroxypregnenolone sulfate, and 2,8-dihydroxyquinoline-beta-D-glucuronide decreased significantly in the acute blood stasis model rats compared with the normal ones.

After administration of the four herb pairs associated with Cyperi Rhizoma, the above potential markers showed different levels of recovery to that in the normal group (*p* > 0.05), except lysoPE(22:4/0:0), lysoPC(17:0), tauroursocholic acid, chimonanthine, cofaryloside, PC(19:3/0:0), and uralenneoside.

In the four administration herb pairs group, the four compounds including trihydroxycholan-24-oic acid, arachidonic acid, indoxylsulfuric acid, and 17-hydroxypregnenolone sulfate showed significantly change compared with the model group (*p* < 0.05). While compared with the model group, the concentration of lysoPE(20:2/0:0) increased significantly in XD and XC group, taurodeoxycholic acid decreased in XB group, lysoPE(22:1/0:0) increased significantly in XB and XY group, estrone 3-glucuronide decreased significantly in XC and XB group, 5-(4-acetoxybut-1-ynyl)-2,2′-bithiophene decreased significantly in XD, XB, and XY group, polyethylene oxidized decreased significantly in XD group, succinoadenosine decreased significantly in XC and XB group, 2, 8-dihydroxyquinoline-β-D-glucuronide increased significantly in XC and XB group XD and XB group (*p* < 0.05).

**Figure 4:**
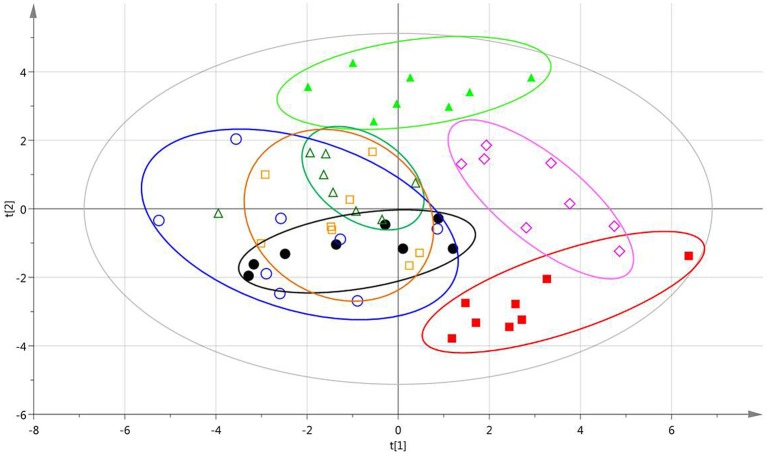
PCA scores plots of samples based on the relative concentrations of 23 endogenous metabolites. Normal group (red 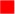), Model group (bright green 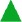), Aspirin group (black 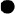), XD group (amaranth 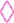), XC group (saffron yellow 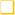), XB group (blue 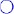), XY group (bottle green 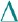).

After data alignment, normalization and unit variance scaling, PCA score plots based on the relative concentrations of 23 endogenous metabolites revealed a trend of intergroup separation and intragroup aggregation (Figure 4). The relative distance of XD, XC, XB, and XY groups separated from the model group with a trend near to the normal group, indicating that XD, XC, XB, and XY groups were able to adjust the abnormal metabolism of the acute blood stasis rats to the normal state. Moreover, the relative distance of XD group showed closer to the normal group, which suggested that XD group had better effect of blood circulation and dissipating blood stasis than the others.

### Gene expression analysis

**Figure 5:**
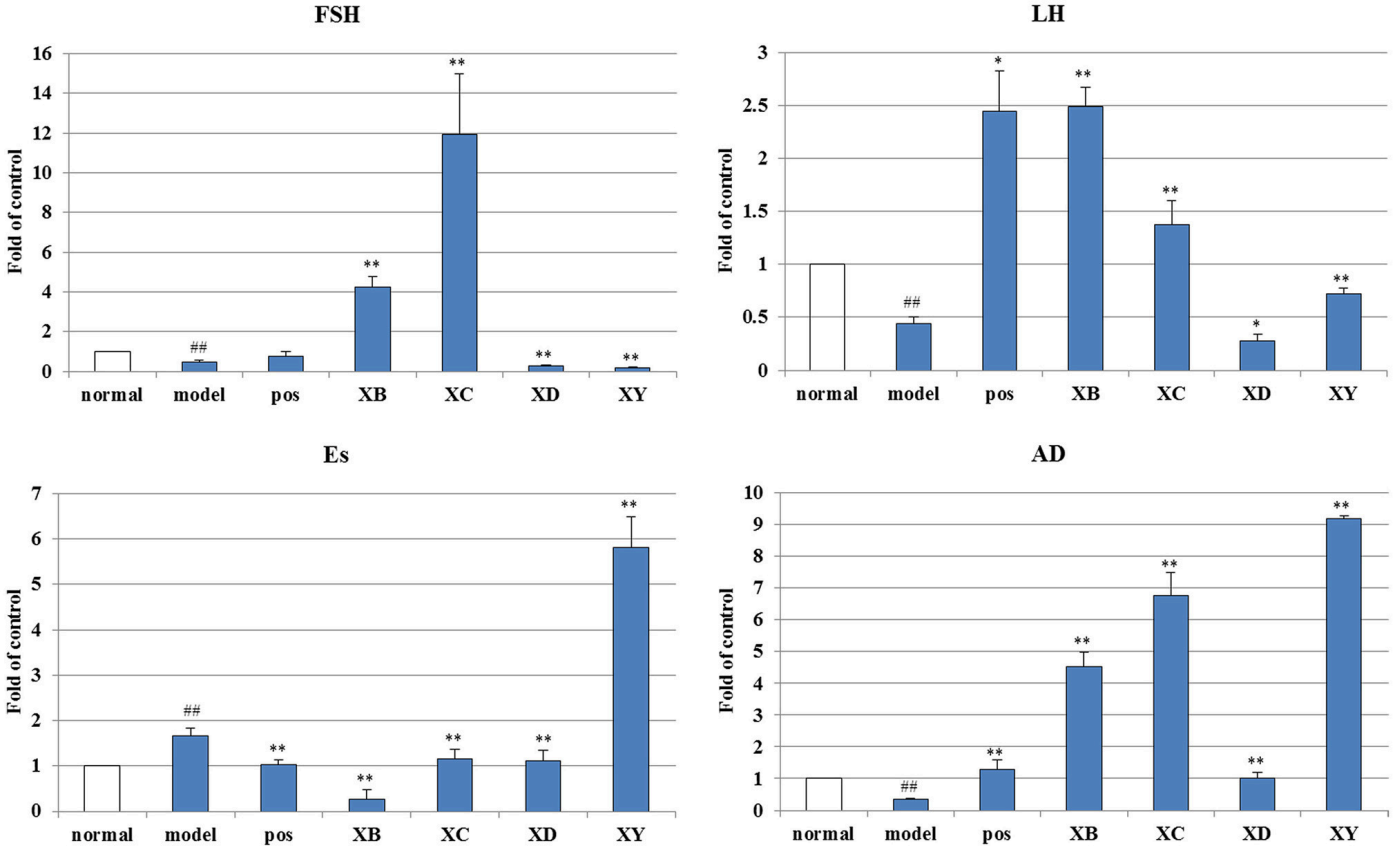
The effects of Cyperi Rhizoma series of herb pairs treatment on endocrine gene expression in acute blood stasis model rats. Gene expression was measured by *q*PCR (*n* = 8 per group). ^##^*p* < 0.01 compared to the normal group, ^*^p < 0.05, ^**^p < 0.01 compared to the model group.

mRNA expression levels of FSH and luteinizing hormone (LH) in the ovary, androgen (AD) and estrogen (Es) in the uterus were analyzed by quantitative polymerase chain reaction (*q*PCR) (Figure 5).

For enzymes related to endocrine, the mRNA levels of FSH (0.46 of the normal group, compared with the normal group, *p* < 0.01), LH (0.44 of the normal group, compared with the normal group, *p* < 0.01), and AD (0.33 of the normal group, compared with the normal group, *p* < 0.01) significantly decreased while increased Es (1.66 of the normal group, compared with the normal group, *p* < 0.01).

Cyperi Rhizoma series of herb pairs regulated the endocrine gene expression in acute blood stasis model rats. XB and XC up-regulated expressions of FSH (4.23 and 11.95 of the normal group, compared with the model group, *p* < 0.01). XB, XC, and XY up-regulated expressions of LH (2.49, 1.38, and 0.72 of the normal group, compared with the model group, *p* < 0.05). XB, XC, XD, and XY up-regulated expressions of AD (4.53, 6.76, 1.01, and 9.20 of the normal group, compared with the model group, *p* < 0.01). XB, XC, and XD down-regulated expressions of Es (0.25, 1.15, and 1.12 of the normal group, compared with the model group, *p* < 0.01).

### Correlation analysis between the markers, biochemistry indicators, and gene expression

In the experiment, several factors, including different biochemistry factors, potential markers, and mRNA were tested. To monitor changes in a whole suite of these factors, correlation networks method was used here. The correlations among these important substances were found based on the alteration of all test factors by building correlation networks. According to the reference method (Liu et al., 2013c), the substances were connected according to their Pearson correlation coefficient (*r*) and the significance of the connection was set at the *p* < 0.05 level.

**Figure 6:**
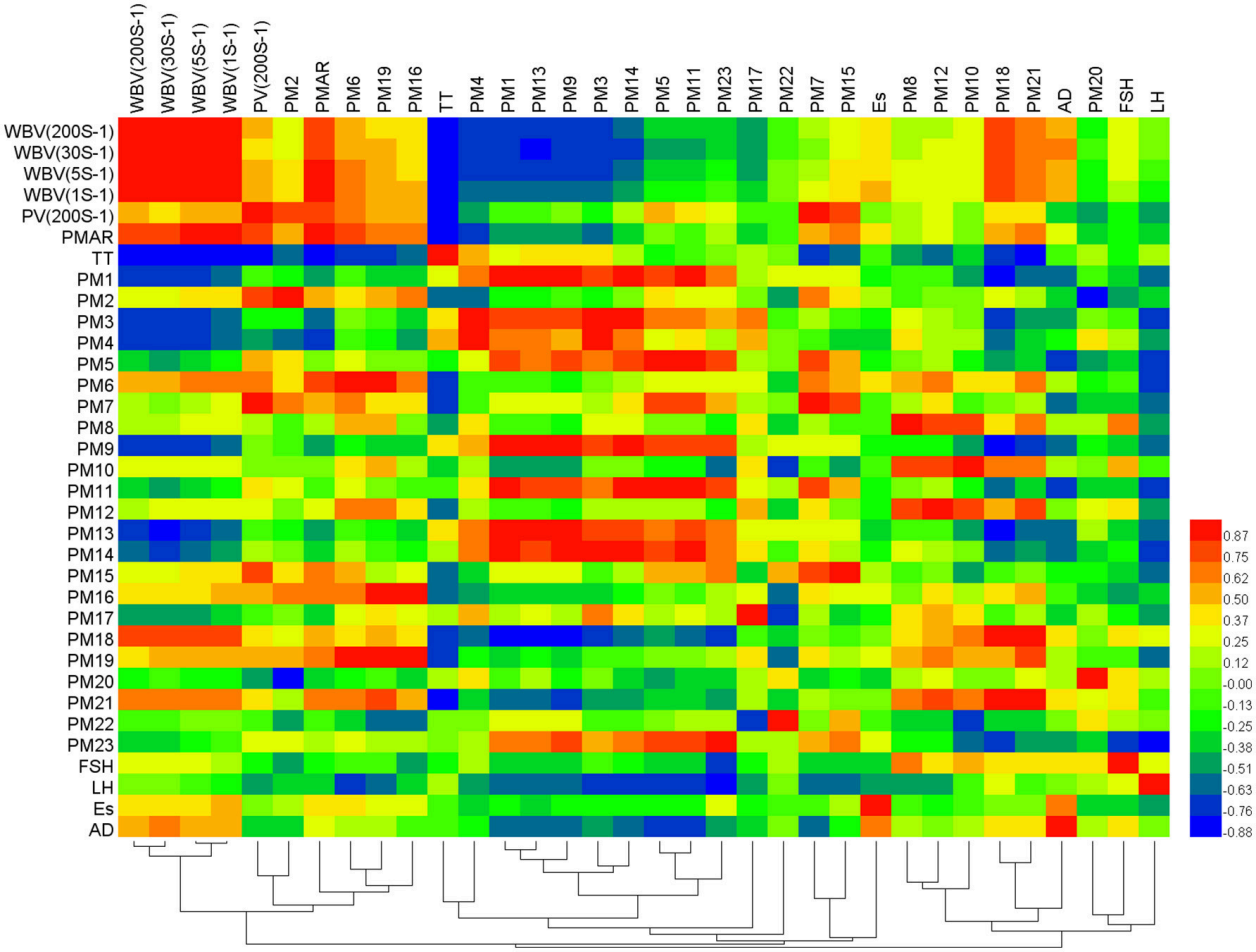
Correlation analysis between biochemistry factors, potential markers, and mRNA. Color key indicates correlated value, blue: negatively correlated, red: positively correlated.

A correlation heat-map showed the systemic substance changes of blood stasis condition and Cyperi Rhizoma series of herb pairs intervention. As shown in Figure 6, hemorheology indexes, WBV(200S^−1^), WBV(30S^−1^), and WBV(5S^−1^), negatively correlated with lysoPE(22:4/0:0), lysoPC(17:0), lysoPE(20:2/0:0), lysoPE(22:1/0:0), and PC(19:3/0:0) (*r* < −0.6), positively correlated with polyethylene oxidized and uralenneoside (*r* > 0.6). WBV(1S^−1^) negatively correlated with lysoPE(20:2/0:0) and PC(19:3/0:0) (*r* = −0.628, −0.609), positively correlated with taurodeoxycholic acid, polyethylene oxidized, succinoadenosine and uralenneoside (*r* = 0.707, 0.750, 0.613, 0.736). PV(200S^−1^) positively correlated with trihydroxycholan-24-oic acid, taurodeoxycholic acid, arachidonic acid, and indoxylsulfuric acid (*r* = 0.814, 0.646, 0.901, 0.766). PMAR negatively correlated with lysoPE(20:2/0:0) (*r* = −0.670), positively correlated with trihydroxycholan-24-oic acid, taurodeoxycholic acid, indoxylsulfuric acid, estrone 3-glucuronide, polyethylene oxidized, succinoadenosine, uralenneoside (*r* = 0.609, 0.770, 0.684, 0.728, 0.609, 0.698, 0.635). TT negatively correlated with taurodeoxycholic acid, arachidonic acid, polyethylene oxidized, succinoadenosine, and uralenneoside (*r* = −0.740, −0.675, −0.739, −0.671, −0.782). FSH mRNA negatively correlated with dihydrocaffeic acid 3-sulfate (*r* = −0.724), positively correlated with tauroursocholic acid and chimonanthine (*r* = 0.674, 0.619). LH mRNA negatively correlated with lysoPC(17:0), lysoPE(24:6/0:0), taurodeoxycholic acid, arachidonic acid, PG(10:0/10:0), lysoPE(0:0/18:0), and dihydrocaffeic acid 3-sulfate (*r* = −0.642, −0.685, −0.725, −0.621, −0.733, −0.690, −0.779). AD mRNA negatively correlated with lysoPE(24:6/0:0), lysoPE(22:1/0:0) and PG(10:0/10:0) (*r* = −0.713, −0.604, −0.648).

### Principal component analysis (PCA) of tested factors

To further evaluate the variations in the biochemistry factors, potential markers and mRNA in all groups, PCA was performed on the basis of the contents of 34 tested factors. In this study, the contents of the 34 tested factors analyzed from the 7 groups composed a data matrix with 7 rows and 34 columns. The first six principal components (PC1, PC2, PC3, PC4, PC5, and PC6) with 100% of the whole variance were extracted for analysis. Among these, PC1, PC2, PC3, PC4, PC5, and PC6 explained 38.347, 25.456, 14.158, 11.430, 5.902, and 4.707% of the total variance, respectively. The factor load matrix is shown in Table 3. F_1_, F_2_, F_3_, F_4_, F_5_, and F_6_ were calculated by the loadings and contents vectors. The full principal components score (F) was then calculated as *F* = 0.38347F_1_+0.25456F_2_+0.14158F_3_+0.11430F_4_+0.05902F_5_+0.04707F_6_. Finally, the *F*-value exhibited an order of Model group (20.72) > XY group (20.30) > XC group (18.24) > XB group (16.69) > XD group (13.26) > Aspirin group (12.78) > Normal group (12.39).

**Table 3:** The factor load matrix (loadings of X).

	**Principal component (PC)**					
	**1**	**2**	**3**	**4**	**5**	**6**
WBV (200 S^−1^)	0.925	0.052	−0.149	0.199	−0.039	0.280
WBV (30 S^−1^)	0.953	−0.016	−0.123	0.229	−0.037	0.147
WBV (5 S^−1^)	0.924	0.134	−0.131	0.266	−0.058	0.193
WBV (1 S^−1^)	0.899	0.230	−0.100	0.316	−0.027	0.168
PV (200 S^−1^)	0.507	0.767	−0.142	−0.215	−0.280	0.098
PMAR	0.798	0.484	−0.268	0.220	−0.084	−0.047
TT	−0.798	−0.500	−0.091	−0.022	0.314	−0.076
PM1	−0.823	0.528	0.055	0.100	−0.039	0.170
PM2	0.465	0.595	−0.302	−0.564	0.123	−0.076
PM3	−0.729	0.421	0.467	0.038	0.258	0.069
PM4	−0.728	0.086	0.611	0.227	0.195	0.002
PM5	−0.396	0.868	0.008	−0.213	−0.130	0.165
PM6	0.559	0.696	0.239	0.286	0.228	−0.108
PM7	0.146	0.930	0.006	−0.133	−0.308	−0.007
PM8	0.297	0.222	0.885	0.152	0.110	0.211
PM9	−0.337	0.003	−0.298	0.888	−0.021	0.093
PM10	0.482	−0.051	0.812	−0.229	0.223	0.055
PM11	−0.484	0.845	0.056	−0.063	−0.131	0.165
PM12	0.388	0.375	0.807	0.077	−0.103	−0.203
PM13	−0.840	0.499	0.121	0.147	−0.095	−0.004
PM14	−0.612	0.644	0.303	−0.174	0.152	0.256
PM15	0.155	0.755	−0.400	0.301	−0.349	0.182
PM16	0.606	0.469	−0.118	−0.116	0.198	−0.589
PM17	0.534	0.001	0.309	−0.728	0.220	0.204
PM18	0.944	−0.145	0.227	−0.122	−0.140	−0.045
PM19	0.622	0.518	0.284	0.132	0.254	−0.427
PM20	−0.167	−0.384	0.427	0.556	−0.435	−0.379
PM21	0.832	0.085	0.463	0.144	−0.145	−0.211
PM22	−0.665	0.253	0.216	0.560	−0.266	−0.250
PM23	−0.423	0.790	−0.368	0.153	0.185	−0.061
FSH	0.258	−0.408	0.662	0.176	−0.247	0.487
LH	0.132	−0.851	−0.168	−0.312	−0.362	−0.040
Es	0.345	0.147	−0.408	0.445	0.696	0.102
AD	0.488	−0.426	0.018	0.646	0.351	0.202

## Discussion

The blood stasis, one kind of pathogenic factors for some diseases, (such as gynecological disorders), is due to poor blood circulation, anesthesia in the vein, or in the over-flow, and the condensation of the pathological products formed in a local (Liu et al., 2012; Han Y. Q. et al., 2016). Our previous studies showed that XFSWD could improve the indexes of rats in the model of acute blood stasis, including WBV, PV, TT, E_2_ and so on (Liu et al., 2010). In TCM, the principle of forming a prescription is implemented with seven different combinations in drug (going alone, mutual reinforcement, mutual assistance, mutual restraint, mutual detoxication, etc). The compatibility of mutual assistance could enhance the efficacy in a prescription. XF series of herb pairs were important mutual assistance compatibility units in XFSWD. XF, in conjunction with DG, CX, BS, or YH, improve the efficacy of promoting blood circulation and removing blood stasis. Why one kind of herb medicine used with different ones could obtain the same efficacy? Did these herb pairs present the same pharmacological activity or action targets? To answer the above questions, we designed the metabolomics experiments.

### Comparative analysis of herb pairs efficacy on invigorating blood circulation

Since the classic of TCM “*Neijing*” described the pathological mechanisms of blood stasis as “blood coagulation and weep,” more than 90% of studies used hemorheology indexes to an animal model establishment and pharmacology evaluations rather than a pathological method (Zhang et al., 2017). In this study, no liver, ovary, and uterus pathological damage occurred in the acute blood stasis model rats, which indicated that acute blood stasis may not cause organic lesions.

Partly because of the easy application, many hemorheology index evaluations of the acute blood stasis have been reported (Liao J. et al., 2016; Zhang et al., 2017). Here, the unified hemorheology index of whole blood viscosity, platelet aggregation rate and TT were used for assessment of rat acute blood stasis models. For the herb pairs treatments, XD achieved the best efficacy with alleviated the unified hemorheology index, PMAR and TT. For DG, butylidenephthalide, ferulic acid, and ligustilide were the main components that be proved to display the blood-activating and stasis-dissolving effect (Yue et al., 2017). While the other three CX, BS, and YH, with the traditional efficacy of promoting Qi circulation to relieve pain, mainly focus on the pharmacological activity including anti-inflammatory and analgesia. So, these observations were in agreement with the theory of the compatibility of XF series of herb pairs, and reflected the rule of compatibility preliminarily.

### Relatively quantitative analysis of herb pairs efficacy based on correlation and PCA

As to the semi-quantitative results of these common differential metabolites, it was especially interesting that trihydroxycholan-24-oic acid (PM2), arachidonic acid (PM7), indoxylsulfuric acid (PM15) and 17-hydroxypregnenolone sulfate (PM20) were related to XD, XB, XC, and XY four treatment groups.

Trihydroxycholan-24-oic acid and 17-hydroxypregnenolone sulfate were steroidal amphipathic molecules. Plasma bile acids were not only known to be essential in dietary lipid absorption and cholesterol catabolism, but also ligands for the G protein-coupled receptor (Watanabe et al., 2006). Trihydroxycholan-24-oic acid was one kind of bile acid. In this experiment, it ralated to PV and PMAR positively, which suggested blood stasis may disturb the cholesterol catabolism, and G protein-coupled receptor could be the potential target of XF series of herb pairs. 17-Hydroxypregnenolone is a prohormone of the sex steroids. The steroid is frequently used as a marker of congenital adrenal hyperplasia and gonadal dysfunction (Vcelakova et al., 2007). Our previous study had reported 17-hydroxypregnenolone was one marker involved in the pathology of primary dysmenorrhea (Liu et al., 2013c). Here, the contents of this sex steroid showed negatively related to trihydroxycholan-24-oic acid, while another marker arachidonic acid (an important inflammation mediator) showed positively related. Moreover, arachidonic acid negatively related to TT and LH, but positively related to indoxylsulfuric acid, one of the most important uremic toxins that could enhance inflammatory response and oxidative stress (Adesso et al., 2017). These results gave a clue that blood stasis had some internal relationship with gynecological disease at metabolites level. Inflammatory factors and endocrine hormones took part in the pathology of blood stasis.

The previous literature has showed that phenylalanine metabolism, sphingolipid metabolism, arachidonic acid metabolism, and arginine and proline metabolism are related to acute blood stasis rats (Li et al., 2015). Here, a significant up-regulation of trihydroxycholan-24-oic acid but down-regulation of 17-hydroxypregnenolone sulfate was found only in model group, implying the acute blood stasis probably disturbed the steroid hormone biosynthesis pathway. It is worth pointing out that, XD, XC, XB, and XY presented different modulatory ability of the metabolites.

The metabolic changes in chemicals exposure could be mapped by mean metabolic position (Nicholson et al., 2002). The selected markers from normal, model and herb pairs treatment groups in PCA trajectory plots were drew. The tendency in PCA score plots for the potential markers was approximately consistent with the herb pairs treatments pharmaceutical effect. It suggested that the selected markers could reflect the effect of intervention. The full PCA scores sorting including biochemistry factors, potential markers and mRNA further showed that XD obtained the best effect, while XY was poor on blood stasis. From the above, we could draw the conclusion that XD, XC, XB, and XY could invigorate blood circulation by altering the glycerophospholipid metabolism, steroid hormone biosynthesis and arachidonic acid metabolism pathway. Although they were all mutual-assistance compatibility, XD was more prominent in regulating the blood stasis from the aspect of global metabolic profile and the specifically altered metabolites, respectively. These results may indicate that the profile of the metabolomics was closely correlated with traditional biological end points, and was thus becoming a useful tool for efficacy evaluation and compatibility discussion.

## Conclusion

In the development of herbal therapy, single herb formulae were gradually evolved into the multi-herb formulae. Herb pairs, two herbs combination, could be an entry point to study the complicated formulae. The present study investigated metabolic variations on invigorating blood circulation for XF series of herb pairs using MS-based metabolomics. These variations involved significant perturbations in glycerophospholipid metabolism, steroid hormone biosynthesis, and arachidonic acid metabolism. Multiple pharmacodynamics index evaluation, relatively quantitative analysis of the metabolic markers, multivariate statistical analysis of effect index, metabolites and genes showed distinct changes in different compatibility. Collectively, these results indicated that the four herb pairs, XD, XC, XB, and XY, exerted effect on the treatment of adrenaline hydrochloride and ice-water bath induced acute blood-stasis rats. However, not only the efficacy extent, but also the global metabolic alteration was difference. Cyperi Rhizoma—Angelicae Sinensis Radix (XD) played a crucial role in XFSWD on invigorating blood circulation. Consequently, the results of this study may provide some heuristic guidance for TCM formulation rationalization and optimization.

## Author contributions

PL carried out most of the studies, performed statistical analysis and wrote the manuscript. YZ and JY participated in the animal and PCR experiments. ES and DQ participated in the data processing work. PL and JD conceived and designed the experiments, revised the manuscript. All authors have read and approved the final version.

### Conflict of interest statement

The authors declare that the research was conducted in the absence of any commercial or financial relationships that could be construed as a potential conflict of interest.

## Abbreviations

AD: Androgen
ADP: Adenosine diphosphate
ANOVA: analysis of variance
BS: Paeoniae Radix Alba
CX: Chuanxiong Rhizoma
DG: Angelicae Sinensis Radix
Es: estrogen
ESI: electrospray ionization
FSH: follicle-stimulating hormone
HCA: hierarchical cluster analysis
LC-MS: liquid chromatography-mass spectrometry
LH: luteinizing hormone
MS: mass spectrometry
OPLS-DA: orthogonal partial least-squared discriminant analysis
PCA: principal components analysis
PLS-DA: partial least squares discriminant analysis
PMAR: ADP-induced platelet maximum aggregation
PV: plasma viscosity
*q*PCR: quantitative polymerase chain reaction
TCM: traditional Chinese medicine
TT: thrombin time
UPLC-MS/MS: Ultra Performance Liquid Chromatography- tandem mass spectrometry
VIP: variable importance in the projection
WBV: whole blood viscosity
XB: Cyperi Rhizoma—Paeoniae Radix Alba
XC: Cyperi Rhizoma—Chuanxiong Rhizoma
XD: Cyperi Rhizoma—Angelicae Sinensis Radix
XF: Cyperi Rhizoma
XFSWD: Xiang-Fu-Si-Wu Decoction
XY: Cyperi Rhizoma—Corydalis Rhizoma
YH: Corydalis Rhizoma.
