# Supplementary material for: Comparative Analysis of Compatibility Effects on Invigorating Blood Circulation for Cyperi Rhizoma Series of Herb Pairs Using Untargeted Metabolomics
Source: Front Pharmacol. 2017 Sep 26;8:677. doi: 10.3389/fphar.2017.00677 (PMC5622986; doi:10.3389/fphar.2017.00677)
Supplement: Supplementary file 1 [file Presentation1.pdf]

The process of sample preparation

Plasma and urine samples were thawed at room temperature before preparation. Acetonitrile (600 μL) was added into each plasma sample (200 μL) to precipitate protein. Afterwards, the mixture was vortexed for 30 s and centrifuged at 3000 rpm for 10 min. Acetonitrile (800 μL) was added into 800 μL of urine sample, the mixture was vortexed for 1 min and centrifuged at 13000 rpm for 10 min. Then, 450 μL supernatants of the plasma samples and 1400 μL supernatants of the urine samples were transferred into new tubes and evaporated to dryness under vacuum with the Lab-conco CentriVap concentrator (Kansas City, MO, USA), respectively. The residues of plasma and urine samples were dissolved in 200 μL of 70% acetonitrile solution, and the mixtures were vortexed for 1 min and centrifuged at 13000 rpm for 10 min at 4°C. Finally, a 5 μL aliquot of supernatant was injected for UPLC-MS/MS analysis. In addition, the plasma (or urine) samples were randomly selected from each group and mixed together as the quality control (QC) samples, respectively. This pooled sample was used to provide a representative mean sample containing all analytes that was encountered during the analysis, and it was used to validate stability of LC-MS system. The QC samples were injected five times at the beginning of the run in order to condition or equilibrate the system and then every ten samples to further monitor the stability of the analysis. The acquired QC data were used to investigate the analytical variability in the whole run. This was necessary in order to evaluate whether the analytical system had changed (and to what extent) over the time course of the analysis, and essential for evaluating the variation in the analytical

results and therefore the reliability of the metabolite profiling data (Li et al., 2015).

Figure S1 Representative cross sections of liver, ovary and uterus sections from each group of rats.

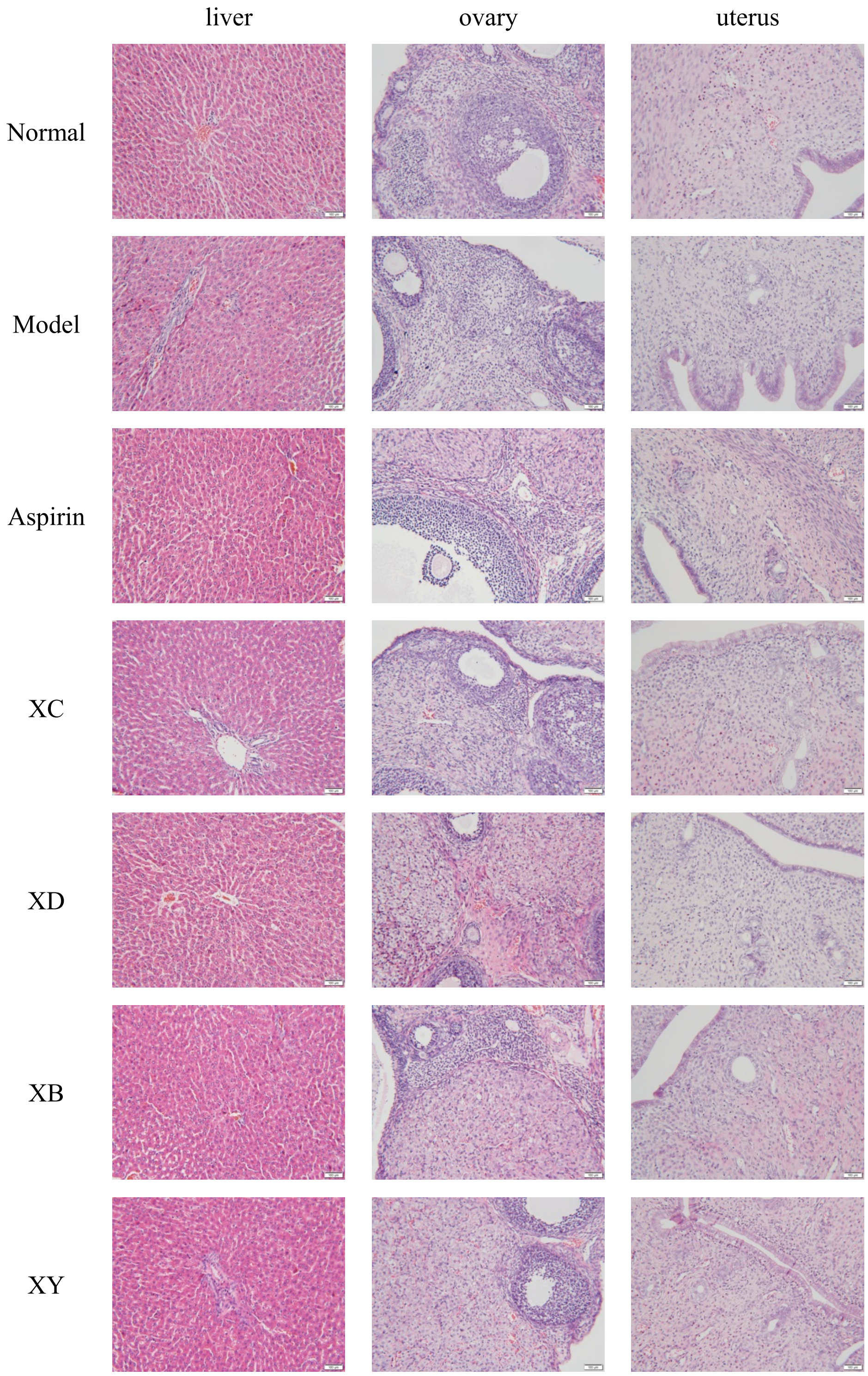

Figure S2 Representative TIC chromatogram of plasma samples at negative ESI mode (I) and positive ESI mode (II) derived from UPLC-Q-TOF/MS. (A) Normal group. (B) Model group. (C) Aspirin group. (D) XD group. (E) XC group. (F) XB group. (G) XY group.

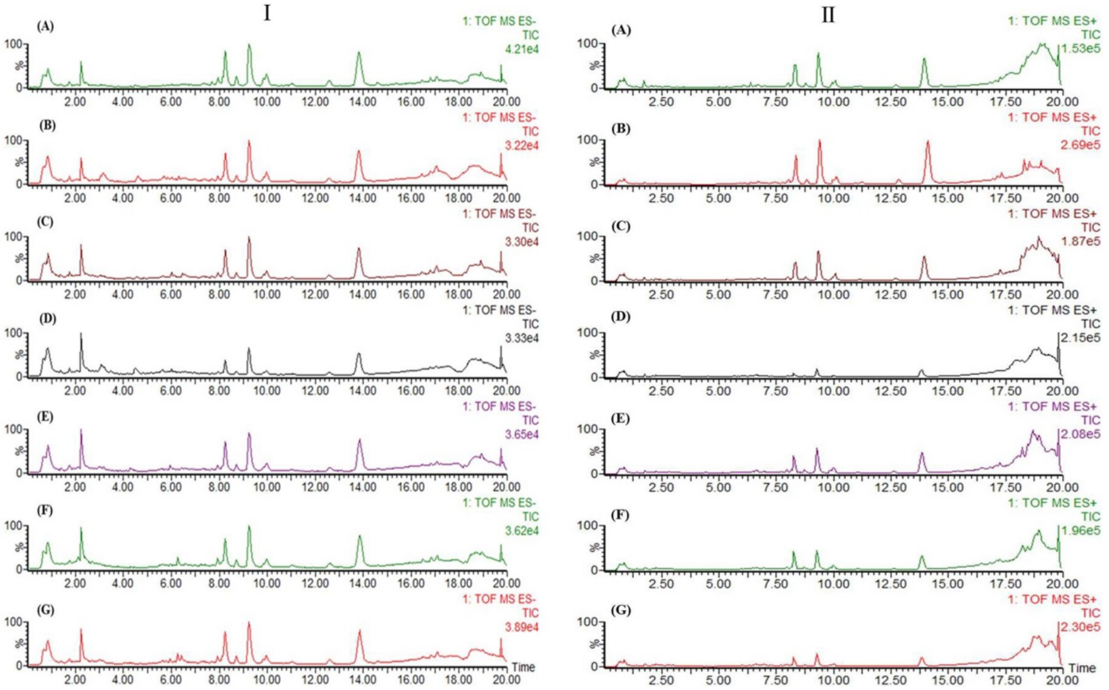

Figure S3 Representative TIC chromatogram of urine samples at negative ESI mode (I) and positive ESI mode (II) derived from UPLC-Q-TOF/MS. (A) Normal group. (B) Model group. (C) Aspirin group. (D) XD group. (E) XC group. (F) XB group. (G) XY group.

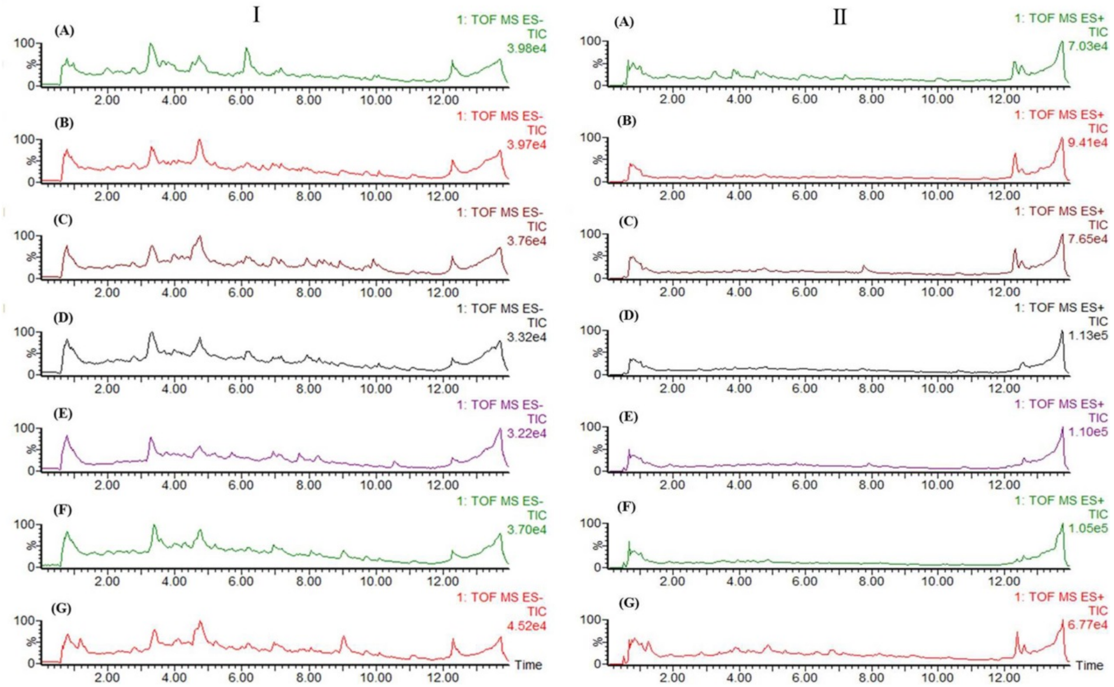

Figure S4 Summary of pathway analysis of plasma and urine samples of the acute blood stasis rats by MetaboAnalyst. a. Arachidonic acid metabolism; b. Steroid hormone biosynthesis; c. Glycerophospholipid metabolism; d. Starch and sucrose metabolism; e. Glycerolipid metabolism; f. Pentose and glucuronate interconversions; g. Linoleic acid metabolism; h. alpha-Linolenic acid metabolism; i. Metabolism of xenobiotics by cytochrome P450; j. Tryptophan metabolism; k. Biosynthesis of unsaturated fatty acids.

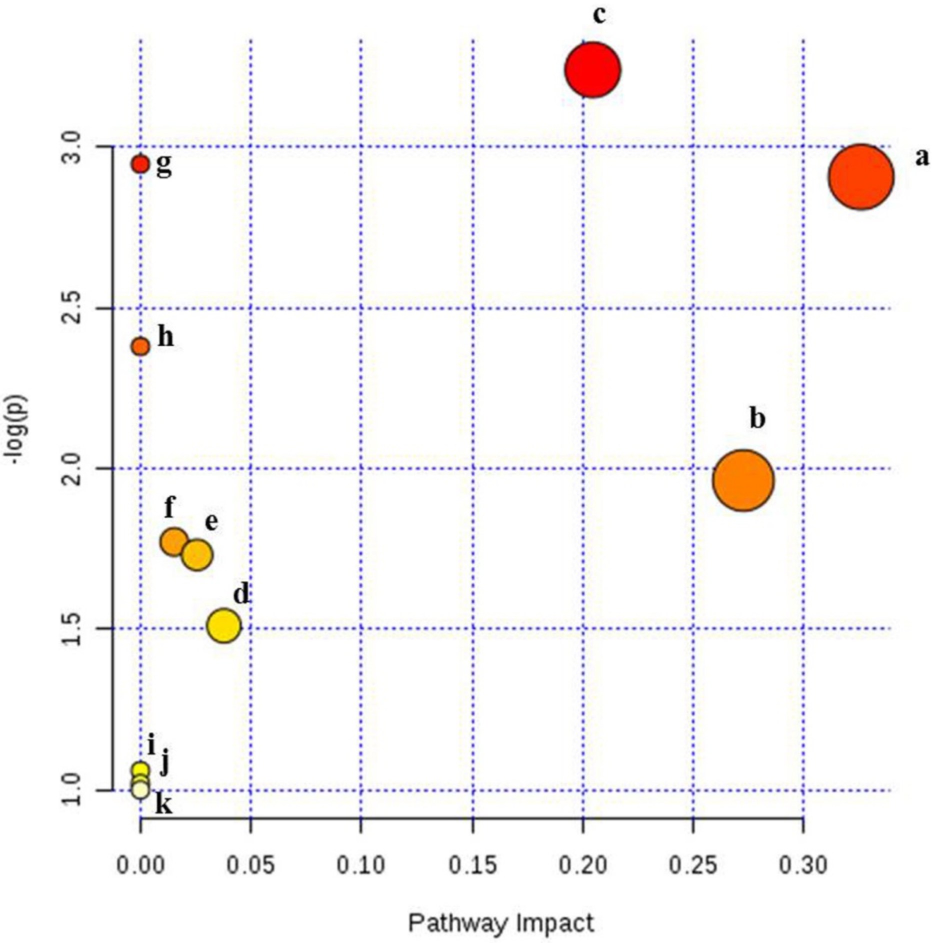

Table S1 Changes in the relative peak area (vs. IS) of target metabolites identified in plasma and urine. (mean ± SD, n=8). $^{\#}p < 0.05$, $^{\#\#}p < 0.01$ compared to the normal group. $^{*}p < 0.05$, $^{**}p < 0.01$ compared to the model group. PM1, lysoPE(22:4/0:0); PM2, trihydroxycholan-24-oic acid; PM3, lysoPC(17:0); PM4, lysoPE(20:2/0:0); PM5, lysoPE(24:6/0:0); PM6, taurodeoxycholic acid; PM7, arachidonic acid; PM8, tauroursocholic acid; PM9, lysoPE(22:1/0:0); PM10, chimonanthine; PM11, PG(10:0/10:0); PM12, cofaryloside; PM13, PC(19:3/0:0); PM14, lysoPE(0:0/18:0); PM15, indoxylsulfuric acid; PM16, estrone 3-glucuronide; PM17, 5-(4-acetoxybut-1-ynyl)-2,2'-bithiophene; PM18, polyethylene oxidized; PM19, succinoadenosine; PM20, 17-hydroxypregnenolone sulfate; PM21, uralenneoside; PM22, 2,8-dihydroxyquinoline-beta-D-glucuronide; PM23, dihydrocaffeic acid 3-sulfate.

No.	Group						
	**Normal**	**Model**	**Aspirin**	**XD**	**XC**	**XB**	**XY**
PM1	1.716 ± 0.241	1.287 ± 0.460$^{\#}$	1.067 ± 0.240	1.473 ± 0.570	1.161 ± 0.282	0.987 ± 0.371	1.091 ± 0.237
PM2	0.061 ± 0.022	0.654 ± 0.500$^{\#\#}$	0.401 ± 0.295	0.216 ± 0.193*	0.073 ± 0.046**	0.087 ± 0.024**	0.276 ± 0.056*
PM3	7.237 ± 0.742	6.136 ± 1.340$^{\#}$	5.889 ± 1.198	7.705 ± 2.339	6.590 ± 1.532	5.259 ± 1.561	5.857 ± 0.979
PM4	0.313 ± 0.074	0.207 ± 0.088$^{\#}$	0.224 ± 0.128	0.350 ± 0.150*	0.299 ± 0.064*	0.224 ± 0.089	0.230 ± 0.066
PM5	0.565 ± 0.113	0.612 ± 0.177	0.386 ± 0.149**	0.514 ± 0.228	0.388 ± 0.131**	0.306 ± 0.124**	0.353 ± 0.079**
PM6	0.073 ± 0.032	0.163 ± 0.071$^{\#\#}$	0.068 ± 0.031**	0.135 ± 0.088	0.121 ± 0.082	0.095 ± 0.047*	0.144 ± 0.097
PM7	0.576 ± 0.160	0.975 ± 0.243$^{\#\#}$	0.404 ± 0.200**	0.620 ± 0.186**	0.477 ± 0.148**	0.472 ± 0.087**	0.460 ± 0.142**
PM8	0.221 ± 0.093	0.407 ± 0.202$^{\#}$	0.218 ± 0.100*	0.474 ± 0.218	0.632 ± 0.446	0.280 ± 0.279	0.313 ± 0.075
PM9	0.385 ± 0.113	0.242 ± 0.074$^{\#\#}$	0.191 ± 0.120	0.278 ± 0.162	0.274 ± 0.060	0.318 ± 0.060**	0.369 ± 0.059*
PM10	0.037 ± 0.029	0.074 ± 0.034$^{\#}$	0.074 ± 0.057	0.084 ± 0.043	0.105 ± 0.072	0.063 ± 0.044	0.063 ± 0.040
PM11	0.200 ± 0.044	0.199 ± 0.070	0.121 ± 0.050*	0.180 ± 0.080	0.136 ± 0.043*	0.108 ± 0.044**	0.124 ± 0.028**
PM12	0.079 ± 0.026	0.148 ± 0.039$^{\#\#}$	0.087 ± 0.048*	0.159 ± 0.089	0.156 ± 0.089	0.125 ± 0.107	0.102 ± 0.059
PM13	0.105 ± 0.040	0.067 ± 0.026$^{\#}$	0.047 ± 0.030	0.093 ± 0.048	0.055 ± 0.016	0.050 ± 0.029	0.049 ± 0.025
PM14	0.789 ± 0.232	0.714 ± 0.192	0.598 ± 0.250	0.786 ± 0.340	0.663 ± 0.179	0.431 ± 0.161**	0.543 ± 0.120*
PM15	11.779 ± 3.329	15.026 ± 1.629$^{\#}$	5.748 ± 2.752**	7.698 ± 1.407**	7.707 ± 3.116**	9.311 ± 2.155**	10.741 ± 3.388**
PM16	0.701 ± 0.343	2.324 ± 0.873$^{\#\#}$	1.541 ± 0.615*	1.955 ± 0.647	1.038 ± 0.431**	1.676 ± 0.289*	1.984 ± 0.532
PM17	0.642 ±	1.548 ±	1.758 ±	1.157 ±	1.587 ±	0.919 ±	1.047 ±

	0.363	$0.350^{\#\#}$	0.581	0.650^{**}	0.259	0.155^{**}	0.371^{**}
PM18	0.191 ± 0.130	0.717 ± $0.130^{\#\#}$	0.592 ± 0.163	0.437 ± 0.060^{**}	0.732 ± 0.106	0.718 ± 0.146	0.594 ± 0.128
PM19	0.155 ± 0.055	0.735 ± $0.180^{\#\#}$	0.335 ± 0.063^{**}	0.702 ± 0.126	0.490 ± 0.091^{**}	0.485 ± 0.071^{**}	0.645 ± 0.136
PM20	0.477 ± 0.184	0.317 ± $0.035^{\#}$	0.310 ± 0.037	0.552 ± 0.066^{**}	0.527 ± 0.075^{**}	0.693 ± 0.056^{**}	0.399 ± 0.152^{**}
PM21	0.661 ± 0.272	1.195 ± $0.149^{\#\#}$	0.886 ± 0.372	1.053 ± 0.165	1.210 ± 0.150	1.192 ± 0.200	1.058 ± 0.152
PM22	0.38 ± 0.137	0.242 ± $0.043^{\#}$	0.157 ± 0.013^{**}	0.368 ± 0.041^{**}	0.252 ± 0.056	0.309 ± 0.040^{**}	0.244 ± 0.025
PM23	1.294 ± 0.344	1.234 ± 0.213	0.836 ± 0.159^{**}	1.179 ± 0.133	0.699 ± 0.123^{**}	0.754 ± 0.134^{**}	1.131 ± 0.168

REFERENCES

Li, S., Lin, H., Tang, Y., Li, W., Shen, J., Kai, J., et al. (2015). Comparative metabolomics analysis on invigorating blood circulation for herb pair Gui-Hong by ultra-high-performance liquid chromatography coupled to quadrupole time-of-flight mass spectrometry and pattern recognition approach. *J Pharm Biomed Anal* 107, 456-463. doi:10.1016/j.jpba.2015.01.029
